# Achillolide A Protects Astrocytes against Oxidative Stress by Reducing Intracellular Reactive Oxygen Species and Interfering with Cell Signaling

**DOI:** 10.3390/molecules21030301

**Published:** 2016-03-02

**Authors:** Anat Elmann, Alona Telerman, Hilla Erlank, Rivka Ofir, Yoel Kashman, Elie Beit-Yannai

**Affiliations:** 1 Department of Food Quality and Safety, The Volcani Center, Agricultural Research Organization, Bet Dagan 50250, Israel; alonat@volcani.agri.gov.il (A.T.); hilla_erlank@walla.com (H.E.); 2 Dead Sea & Arava Science Center and Regenerative Medicine & Stem Cell Research Center, Ben-Gurion University of the Negev, Beer-Sheba 84105, Israel; rivir@bgu.ac.il; 3 Raymond and Beverly Sackler Faculty of Exact Sciences, School of chemistry, Tel Aviv University, Ramat Aviv 69978, Israel; kashman@post.tau.ac.il; 4 Clinical Biochemistry and Pharmacology Department, Faculty of Health Sciences, Ben-Gurion University of the Negev, Beer-Sheba 84105, Israel; bye@bgu.ac.il

**Keywords:** achillolide A, *Achillea fragrantissima*, astrocytes, oxidative stress, reactive oxygen species, mitogen-activated protein kinases (MAPK), neurodegenerative diseases

## Abstract

Achillolide A is a natural sesquiterpene lactone that we have previously shown can inhibit microglial activation. In this study we present evidence for its beneficial effects on astrocytes under oxidative stress, a situation relevant to neurodegenerative diseases and brain injuries. Viability of brain astrocytes (primary cultures) was determined by lactate dehydrogenase (LDH) activity, intracellular ROS levels were detected using 2′,7′-dichlorofluorescein diacetate, *in vitro* antioxidant activity was measured by differential pulse voltammetry, and protein phosphorylation was determined using specific ELISA kits. We have found that achillolide A prevented the H_2_O_2_-induced death of astrocytes, and attenuated the induced intracellular accumulation of reactive oxygen species (ROS). These activities could be attributed to the inhibition of the H_2_O_2_-induced phosphorylation of MAP/ERK kinase 1 (MEK1) and p44/42 mitogen-activated protein kinases (MAPK), and to the antioxidant activity of achillolide A, but not to H_2_O_2_ scavenging. This is the first study that demonstrates its protective effects on brain astrocytes, and its ability to interfere with MAPK activation. We propose that achillolide A deserves further evaluation for its potential to be developed as a drug for the prevention/treatment of neurodegenerative diseases and brain injuries where oxidative stress is part of the pathophysiology.

## 1. Introduction

Oxidative stress is the major underlying contributor to the development of many pathological states, and one of the main risk factors exacerbating neuronal damage in degenerative disorders of the central nervous system that involve different molecular pathways [1,2,3,4,5,6,7]. Oxidative damage and elevated production of H_2_O_2_ in the central nervous system have been implicated in chronic neurodegenerative diseases such as Alzheimer’s disease, Parkinson’s disease, and amyotrophic lateral sclerosis (ALS), as well as in the context of traumatic injuries [5,8,9,10,11]. H_2_O_2_ is a major precursor of highly reactive free radicals. The toxicity of H_2_O_2_, itself is relatively weak compared with that of other active oxygen species, but in the presence of O_2_^−*^, H_2_O_2_ can generate highly reactive hydroxyl radicals via the metal-catalyzed Haber-Weiss reaction. H_2_O_2_ is able to pass through cell membranes, to spread inside and outside of the cell, and to directly and indirectly damage cellular lipids, proteins, and DNA, leading to glial and neuronal cell death. H_2_O_2_ is one of the more stable non-free radical reactive oxygen species (ROS). Therefore H_2_O_2_ may persist for relatively long periods of time to produce neuronal injury [12,13,14,15,16].

In the central nervous system, astrocytes comprise the largest cell population and these cells play multiple roles in the brain. The functions of astrocytes include promoting neuronal survival and plasticity during ischemia and other degenerative injuries, removing toxic materials (e.g., glutamate and free radicals) and providing gliotransmitters to neurons through neuronal-glial interactions [3,17,18,19]. Astrocytes have been implicated in several major neuronal degenerative diseases including Parkinson’s disease, Alzheimer’s disease, and Huntington’s disease [20,21,22]. Despite their antioxidant activity, astrocytes are vulnerable to oxidative stress [23,24,25] and injury can result in impaired astrocyte function, even in cases in which astrocytes do not die. Impaired astrocyte function may critically impair neuronal survival and can amplify neuronal death [18]. Moreover, astrocyte death is observed in brain injuries caused by trauma, ischemia, and neurodegenerative diseases [26,27,28]. Therefore, astrocytes are attractive therapeutic targets. The protection of astrocytes from oxidative stress is essential for the maintenance of brain function, and compounds that possess intrinsic antioxidant properties and/or can trigger the intracellular cascade of protective pathways may represent a promising therapeutic strategy.

*Achillea fragrantissima (Af)* is a desert plant that belongs to the Asteraceae family and has been used for many years in traditional medicine in the Arabian region as a hypoglycemic medicinal plant and for the treatment of respiratory diseases and gastrointestinal disturbances [29,30,31,32,33,34,35]. We have previously shown that achillolide A, a sesquiterpene lactone that we have isolated from *Achillea fragrantissima*, prevents microglial activation, including elevated NO and ROS levels [36]. Sesquiterpene lactones, a group of plant secondary metabolites, are the bioactive compounds in many medicinal plants from the Asteraceae family. These compounds exhibit a broad range of biological activities, including anti-inflammatory, antioxidant, and neuroprotective activities [37,38,39,40].

Since oxidative stress has been accepted as a target of therapeutic interventions for the treatment of brain injuries and neurodegenerative diseases, and in light of the critical role of astrocytes in neuronal survival, in the present study, we evaluated the protective effects of achillolide A against astrocyte death induced by oxidative stress. To elucidate its mechanisms of action, we examined the effects of achillolide A on signal transduction and ROS levels. These activities were compared with those of memantine, a drug used to treat Alzheimer’s disease. The present study provides the first evidence that achillolide A interferes with signaling events and protects astrocytes from oxidative stress.

## 2. Results

### 2.1. Achillolide A Protects Astrocytes against H_2_O_2_-Induced Cell Death

**Figure 1:**
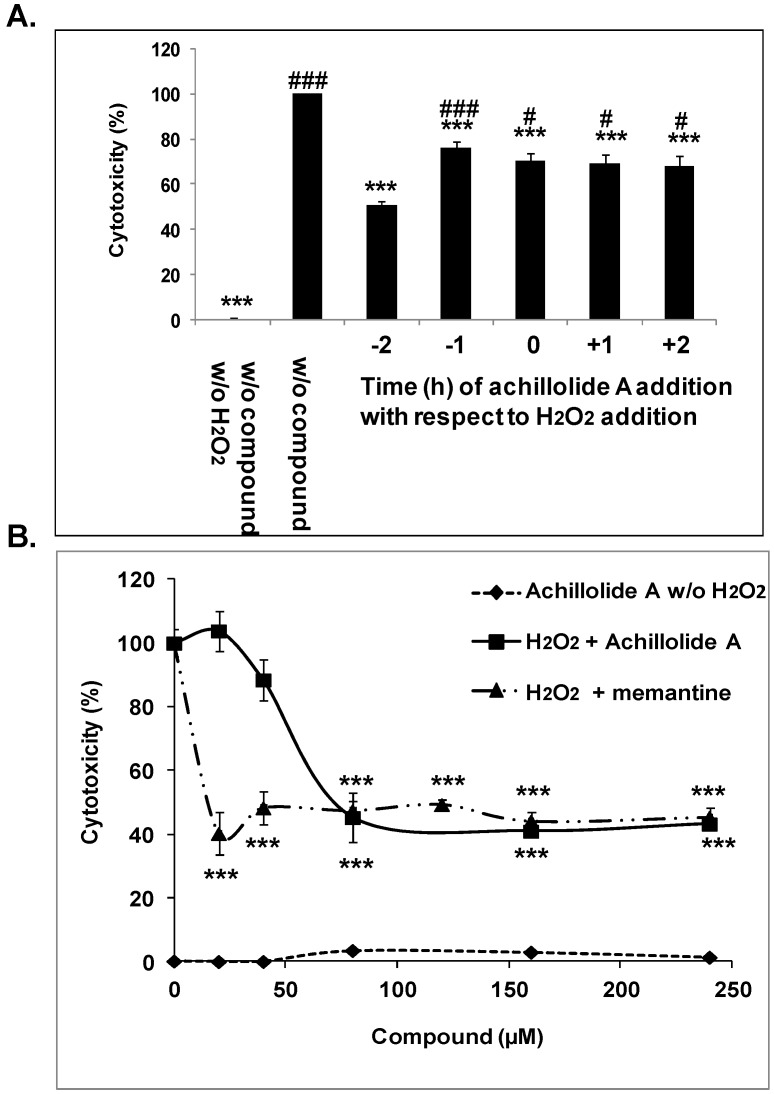
Protective effect of achillolide A on astrocyte cell death induced by H_2_O_2_*.* (**A**) Achillolide A (80 μM) was added to astrocytes before (−2 h, −1 h) concomitant (0) or after (1 h, 2 h) the addition of H_2_O_2_ (175 μM). Cytotoxicity was measured 20 h later by the levels of LDH in the conditioned media. Results are means ± SEM of two experiments (*n* = 8). *^#^* *p* < 0.05, *^###^ p* < 0.001, compared to cells that were preincubated with achillolide A before treatment with H_2_O_2_. *** p < 0.001, compared to cells that were treated with H_2_O_2_ only; (**B**) Astrocytes were pretreated with different concentrations of achillolide A, or memantine (as a control drug). H_2_O_2_ was added 2 h after the addition of compounds and cell death was determined 20 h later by the LDH method. The results are means ± SEM of two experiments (*n* = 7). *** p < 0.001, compared to cells that were treated with H_2_O_2_ only.

Oxidative stress was elicited in cultured primary astrocytes by the addition of 175 μM H_2_O_2_ to the culture medium. The concentration of H_2_O_2_ used in our experiments resembles the concentration reported in rat striatum under ischemic conditions [41]. To assess whether achillolide A can protect astrocytes against oxidative stress, astrocytes were preincubated with achillolide A for 1–2 h before H_2_O_2_ addition, co-treated with H_2_O_2_, or post-treated with H_2_O_2_. Figure 1A shows that achillolide A exhibited a protective effect against H_2_O_2_-induced cell death and that achillolide A is more effective when applied to the cells 2 h before treatment with H_2_O_2_. In order to determine the optimal concentration of achillolide A needed for protection, astrocytes were preincubated with different concentrations of this molecule. Following preincubation, H_2_O_2_ was added, and cytotoxicity was determined 20 h later using the LDH assay. Achillolide A exhibited a protective effect against H_2_O_2_-induced cell death, and was maximally active (55% protection) at 80 μM (Figure 1B). It should be noted, that at all concentrations tested, the cytotoxicity of achillolide A by itself to astrocytes was very low (<3.5%) as determined by the LDH method (Figure 1B). We have compared the protective activity of achillolide A to that of memantine, which is used as a drug for the treatment of Alzheimer’s disease. At the maximal effective concentration of achillolide A (80 μM) as well as at higher concentrations, memantine was similarly effective (*p* > 0.05) to achillolide A and provided 53% protection (Figure 1B). However, it should be noted that memantine was also fully active at lower concentrations (*i.e.*, 20 μM and 40 μM), which were below the active concentrations of achillolide A.

### 2.2. Achillolide A Does Not Have a Hydrogen-Peroxide Scavenging Activity

**Figure 2:**
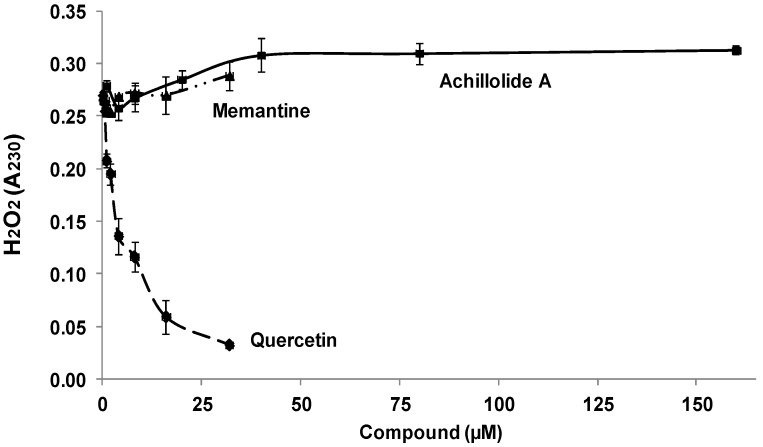
Hydrogen peroxide scavenging ability. For study of H_2_O_2_ scavenging activity, 1 mM H_2_O_2_ and different concentrations of achillolide A, quercetin, or memantine were co-incubated in PBS. Optical density was measured 10 min later. The results are means ± SEM of two experiments performed in duplicates (*n* = 4).

The protective effect of achillolide A against H_2_O_2_ cytotoxicity might be the result of H_2_O_2_ scavenging by achillolide A. We therefore determined the ability of this compound to scavenge H_2_O_2_ in a cell-free assay, and compared it to the scavenging ability of quercetin (which serves as a positive control [42]) and memantine. The results presented in Figure 2 demonstrate that, at all concentrations tested, both achillolide A and memantine did not have any scavenging ability towards H_2_O_2_, while the control flavonoid quercetin scavenged 88% of H_2_O_2_. Thus the protective effect of achillolide A cannot be attributed to H_2_O_2_ scavenging.

### 2.3. Achillolide A Inhibits H_2_O_2_-Induced Phosphorylation of MEK1 and p44/42 MAPK in Astrocytes

The protective effect of achillolide A could be mediated by one mechanism or a combination of several mechanisms: scavenging of H_2_O_2_, scavenging of ROS, and the inhibition of signal-transduction pathways. The first possibility can be excluded because we have shown that achillolide A has no H_2_O_2_ scavenging ability (Figure 2). The last possibility might be supported by the finding that preincubation of astrocytes with achillolide A leads to greater protection than concomitant addition of achillolide A to those cells.

The importance of the MAPK pathway for neurodegenerative diseases and the therapeutic potential of inhibitors of this pathway have been demonstrated in several studies. For example, MEK inhibition has been shown to reduce glial scar formation and promote the recovery of sensorimotor function in rats following spinal cord injury [43]. In addition, inhibition of the ERK signaling pathway was shown to provide neuroprotection in cell models of mechanical trauma [44] and MEK/ERK inhibition reduced microglial activation in a rat model of spinal cord injury [45]. Moreover, p44/42 MAPK has been shown to be activated by oxidative stress and to play a key role in controlling cell apoptosis after oxidative injury [46,47,48]. Specifically, H_2_O_2_ has been reported to stimulate the activity of p44/42 MAPK in primary cultured astrocytes [49]. In light of these previous findings, we attempted to determine whether the protective effect of achillolide A against H_2_O_2_-induced cell death is mediated by the inhibition of H_2_O_2_-induced p44/42 MAPK phosphorylation.

**Figure 3:**
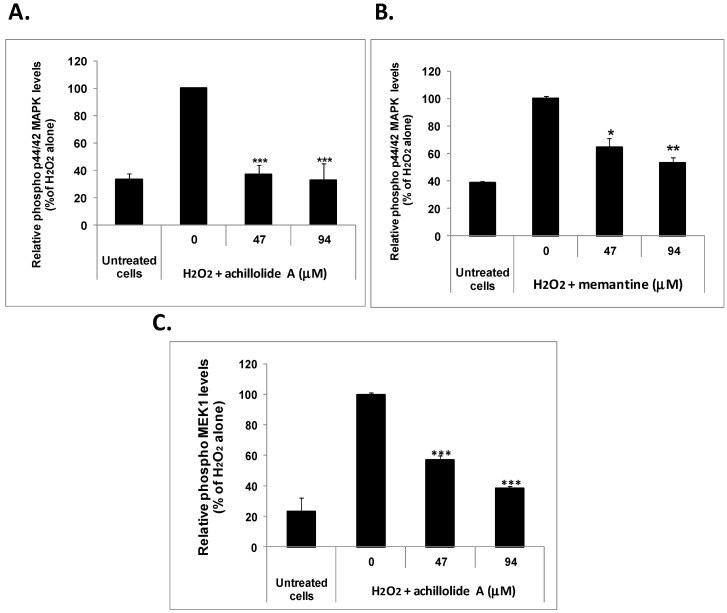
Inhibitory effect of achillolide A on H_2_O_2_-induced phosphorylation of p44/42 MAPK and MEK1 in astrocytes. Astrocytes were treated with 175 μM of H_2_O_2_ for 40 min following preincubation with achillolide A or memantine for 2 h. The levels of phosphorylated and total p44/42 MAPK (**A**,**B**) and phosphorylated and total MEK1 (**C**) were measured by ELISA. Relative phospho MAPK levels were calculated from the ratio: phospho MAPK levels/total MAPK levels the results are means ± SEM of two experiments (*n* = 4). * *p* < 0.05; ** *p* < 0.01; *** *p* < 0.001.

As in the protection experiments described above, astrocytes were pretreated with different concentrations of achillolide A (or memantine as a reference drug) 2 h prior to their exposure to H_2_O_2_ (175 μM) and the phosphorylation of p44/42 MAPK was determined 40 min later in cell homogenates using specific ELISA kits. The results showed that the phosphorylation of p44/42 MAPK was markedly increased by H_2_O_2_ and that achillolide A and memantine each completely inhibit the H_2_O_2_-induced phosphorylation of p44/42 MAPK in astrocytes (Figure 3A,B). Under the same experimental conditions achillolide A also inhibited the H_2_O_2_-induced phosphorylation of MEK1, which is upstream of p44/42 MAPK and is responsible for its phosphorylation (Figure 3C).

We therefore suggest that the protective effects of achillolide A against oxidative stress might be at least partially due to the inhibition of the H_2_O_2_-induced phosphorylation of p44/42 MAPK and MEK1.

### 2.4. Achillolide A Inhibited the H_2_O_2_-Induced Generation of ROS

**Figure 4:**
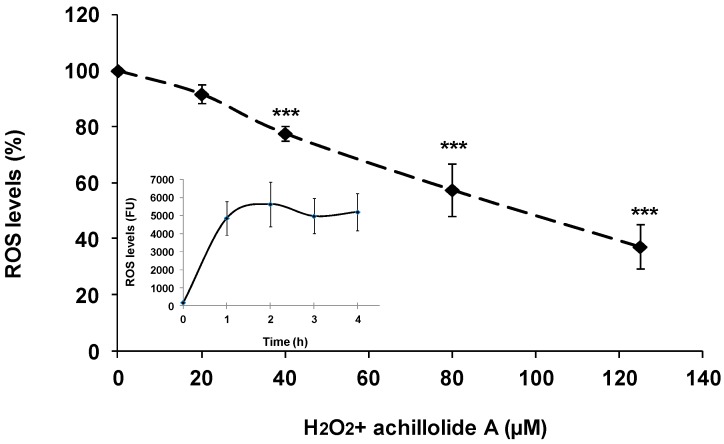
Achillolide A attenuates H_2_O_2_-induced ROS levels in astrocytes. Astrocytes were preloaded with DCF-DA for 30 min and washed. Preloaded astrocytes were then preincubated for 2 h with various concentrations of achillolide A. H_2_O_2_ (175 μM) was added to the culture and the fluorescence intensity representing ROS levels was measured 1 h later. The results represent means ± SEM of two separate experiments (*n* = 8). *** *p* < 0.001. Insert: ROS levels of cells that were treated with 175 μM H_2_O_2_ were measured at the indicated time points.

In our previous study, we demonstrated in a cell-free system that achillolide A is a free radical scavenger with 60% scavenging ability of DPPH, which is a super oxide radical generator [36]. It was therefore reasonable to assume that, in addition to interference with signaling events, achillolide A could protect astrocytes from H_2_O_2_-induced cell death by lowering ROS levels that are induced indirectly by H_2_O_2_. To assess the intracellular levels of ROS, astrocytes were pre-loaded with the ROS indicator 2′,7′-dichlorofluorescein diacetate (DCF-DA), and were pretreated with various concentrations of achillolide A 2 h before H_2_O_2_ insult. ROS formation was determined by examining fluorescence over time. As shown in Figure 4, H_2_O_2_ caused the elevation of intracellular ROS levels (see insert), while pre-treatment of astrocytes with achillolide A inhibited by 65% the levels of intracellular ROS that were induced by H_2_O_2_. Thus, the protective activity of achillolide A might be partially attributed to the reduction in H_2_O_2_-induced intracellular ROS levels.

### 2.5. Differential Pulse Voltammetry (DPV) Analysis of the Antioxidant Capacity of Achilloide A

One common feature of all low-molecular-weight antioxidants is their reducing capability, that is, their ability to donate an electron under suitable conditions. The electrochemical approach for evaluating antioxidant capacity includes measurement of the current that results from oxidation or reduction on an electrode surface following an applied potential difference. The DPV technique has an excellent resolving power and can differentiate between peaks formed by different electroactive species in the same solution that are no more than 50 mV apart [50,51].

In the present study, we used the DPV approach to analyze the total reducing capacities (*i.e.*, the antioxidant capacities) of achillolide A and memantine. As the DPV analysis was done in the absence of astrocytes, the concentrations of achillolide A and memantine (250 μM) were chosen to allow easy demonstration of the antioxidant capacity. Voltammetry analysis results include peaks for which the electric potential (x-axis) value corresponds to the nature of the antioxidant and the current (y-axis) value corresponds to its concentration in the tested sample. A smaller electric potential indicates a more powerful antioxidant that can reduce at a lower electric potential. A higher value for the current indicates a larger amount of the antioxidant [52].

**Figure 5:**
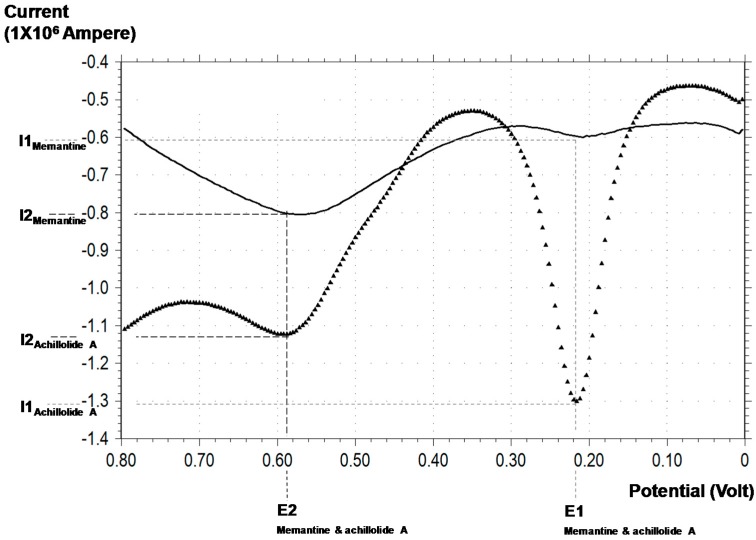
Representative voltammogram of achillolide A and memantine analyses. Representative differential pulse voltammetry (DPV) of aqueous solutions (250 μM) of achillolide A (▲) and memantine (−) with glassy carbon working electrode, Ag/AgCl reference electrode, and Pt wire as counter electrode. E1-First anodic potential, E2-Second anodic potential, I1-First anodic current, and I2-second anodic current.

Voltammetric analysis of achillolide A revealed two peaks, suggesting two electron donations. The E1 peak represents a more potent reducing electron donation at 219 ± 3.83 mV, and the E2 peak represents the less potent electron donation at 589 ± 10.14 mV (Figure 5). Under our experimental conditions, the antioxidant concentration of achillolide A at E1 was slightly higher than that observed for the E2 peak, as represented by the corresponding current values of I1: 1.29 ± 0.064 μA and I2: 1.13 ± 0.08 μA, respectively. Analysis of a memantine solution at the same concentration (250 μM) revealed a significantly lower reducing capacity, indicating that memantine is a weaker antioxidant. The E1 and E2 peaks for memantine were 208 ± 0.00 and 584 ± 13.86 mV, respectively, similar to the E1 and E2 peaks for achillolide A. The similarity of the anodic potentials of the two compounds suggests similar antioxidant potency. The measured current values, I1 and I2, were significantly lower for memantine as compared to achillolide A (0.5911 ± 0.0207 μA and 0.8036 ± 0.0016 μA, respectively), indicating that the antioxidant capacity of memantine is lower than that of achillolide A (Figure 5).

## 3. Discussion

In the present study, we evaluated the ability of achillolide A to counteract oxidative damage in astrocytes. We found that achillolide A protected astrocytes from H_2_O_2_-induced cell death and attenuated the intracellular accumulation of ROS following treatment with H_2_O_2_. Achillolide A also exhibited a broad antioxidant capacity as was demonstrated in the differential pulse voltammetry assay. Moreover, our results indicated that the protective effect of achillolide A against H_2_O_2_-induced cytotoxicity cannot be attributed to direct H_2_O_2_ scavenging but rather to the scavenging of free radicals generated indirectly by H_2_O_2_ and to interference with H_2_O_2_-induced MAPK signaling. The antioxidant capacity of achillolide A was previously demonstrated by our laboratory in a cell-free DPPH assay and by quenching intracellular free radicals produced by 2,2′-azobis(amidinopropane) (ABAP) in cultured microglial cells [36]. Using the electrochemical technique, we were able to demonstrate the powerful reducing capacity of achillolide A—a potential of 220 mV in a hydrophilic environment—similar to ascorbic acid. The second potential (E2) has a value similar to that observed for many polyphenols [53,54], despite the fact that achillolide A is a sesquiterpene lactone and not a polyphenol.

Memantine and achillolide A had similar E1 and E2 values (208; 219 mV and 584; 589 mV, respectively). The DPV resolution is accepted to be about 50 mV, suggesting that these two molecules have similar antioxidant potentials. However, the current measured in the 0.25 mM aqueous solutions of memantine and achillolide A revealed a significant difference in their antioxidant content at I1 (*p* < 0.001) and I2 (*p* < 0.001), indicating that the antioxidant capacity of achillolide A is greater than that of memantine.

The mechanistic basis of the cellular effects of antioxidants does not rely merely on free-radical scavenging or antioxidant activity *per se* [55], but also on the modulation of various signaling events. For example, sesquiterpene lactones have been shown to increase cellular resistance to oxidant injury caused by H_2_O_2_, through Nrf2/ARE-dependent HO-1 expression [56,57]. According to our results, in addition to its antioxidant activity, the sesquiterpene lactone achillolide A, exerts its protective effects on brain astrocytes under oxidative stress through the inhibition of the H_2_O_2_-induced phosphorylation of MEK1 and p44/42 MAPK, which was previously shown to be involved in the response of astrocytes to oxidative stress [49].

It has been proposed [58] that there is a need for new drug regimen strategies based on combinations of memantine and molecules that have antioxidant effects, to create a multi-target therapy to increase neuronal protection and prevent disease progression. According to the results presented in this study, the maximal protective activity of achillolide A against oxidative stress was equal to that of memantine. Moreover, while memantine was a much less potent antioxidant (as was shown by the DPV assay) and could scavenge neither H_2_O_2_ nor free radicals, achillolide A exhibited excellent antioxidant activity and free radical scavenging ability. Thus, achillolide A has activities complementary to those of memantine and the potential additive/synergistic effects of combinations of these substances merit further study.

In a previous study, we demonstrated that achillolide A inhibits microglial activation as manifested by inhibition of the LPS-induced levels of pro-inflammatory and toxic mediators including glutamate, nitric oxide (NO), matrix metalloproteinase-9 (MMP-9), cyclooxygenase-2 (COX-2), induced nitric oxide synthase (iNOS), interleukin-1 beta (IL-1 β), and tumor necrosis factor-alpha (TNF-α) [36]. Achillolide A also exhibits antioxidant activity, as was shown in a cell-free system and by its ability to reduce intracellular ROS levels in microglial cells.

Since achillolide A can inhibit microglial activation, protect astrocytes from oxidative stress, modulate MAPK activities, and reduce ROS levels, it is proposed that achillolide A deserves further evaluation of its potential to be developed as a drug for the treatment of neurodegenerative diseases in which inflammation, oxidative stress and astrocytic cell death play important roles.

## 4. Materials and Methods

### 4.1. Materials

Dulbecco’s modified Eagle’s medium (DMEM), Leibovitz-15 medium, glutamine, antibiotics (10,000 IU/mL penicillin and 10,000 μg/mL streptomycin), soybean trypsin inhibitor, fetal bovine serum (FBS), and Dulbecco’s phosphate buffered saline (PBS) (without calcium and magnesium) were purchased from Biological Industries (Beit Haemek, Israel); memantine, 2,2-Diphenyl-1-picrylhydrazyl (DPPH), and 2′,7′-dichlorofluorescein diacetate (DCF-DA) were purchased from Sigma Chemical Co. (St. Louis, MO, USA). Dimethyl sulfoxide (DMSO) was obtained from Applichem (Darmstadt, Germany); and hydrogen peroxide (H_2_O_2_) was obtained from MP Biomedicals (Solon, OH, USA).

### 4.2. Plant Material

The aerial parts of *Achillea fragrantissima* were collected in the Arava Valley, and the voucher specimens have been kept and authenticated as part of the Arava Rift Valley Plant Collectionunder the accession code AVPC0040.

### 4.3. Purification of Achillolide A

The dry aerial parts of *Achillea fragrantissima* (37 g) were homogenized and extracted with ethyl acetate (EA; 3 × 100 mL). Evaporation of the EA gave a brown gum (2.5 g) that was chromatographed on Sephdex LH-20 (2.5 cm × 30 cm) and eluted with petroleum ether/CH_2_Cl_2_/MeOH (2:1:1), 300 mL; 10 fractions of 30 mL. Fractions containing achillolide A (TLC, silica, eluted with EA/petrol ether 1:1, R_f_ 0.5) were combined and evaporated (under vacuum on a Buchi rotavapor, 9230 Flawil, Switzerland), to give crude achillolide A, 290 mg. The latter was re-chromatographed by vacuum liquid chromatography (VLC) on silica gel (2 cm × 5 cm column bed) eluted with petrol ether EA of increasing polarity (the ethyl acetate percentages was raised by 5% at a time); 15 fractions of 25 mL. Achillolide A (90 mg) was obtained from fraction eluted with 30% EA by evaporation of the solvent. Crystallization from petrol ether/acetone mixture (prepared by volume), performed twice, gave pure (98%) achillolide A (40 mg), as was determined by NMR and according to the melting point and optical activity.

### 4.4. Preparation of Primary Cultures of Astrocytes

Primary cultures of astrocytes were prepared from cerebral cortices of one- or two-day-old neonatal Wistar rats. Briefly, meninges and blood vessels were carefully removed from cerebral cortices and kept in a Leibovitz-15 medium; brain tissues were dissociated by trypsinization with 0.5% trypsin (10 min, 37 °C, 5% CO_2_); and cells were washed first with DMEM containing soybean trypsin inhibitor (100 μg/mL) and 10% FBS and then with DMEM containing 10% FBS. Cells were seeded in tissue culture flasks pre-coated with poly-d-lysine (20 μg/mL in 0.1 M borate buffer pH 8.4) and incubated at 37 °C in humidified air with 5% CO_2_. The medium was changed on the second day and every second day thereafter. At the time of primary cell confluence, microglial and progenitor cells were discarded by shaking (180 RPM, 37 °C, 6 h + 18 h) the flasks on a horizontal shaking platform. Twenty-four hours later, cytosine β-d-arabinofuranoside was added for 24 h. The medium was then changed and 14- to 17-day-old astrocytes were used in the experiments. The research was conducted in accordance with the NIH guide for the care and use of laboratory animals, and was approved by the Institutional Animal Care and Use committee of the Volcani institute, Agricultural Research Organization (IL-135/07, approval date 04.11.07).

### 4.5. Treatment of Astrocytes with H_2_O_2_

Astrocytes were re-plated on 24-well poly-d-lysine-coated (PDL) plastic plates, at a density of 1 × 10^5^/well, in DMEM (without phenol red) containing 2% FBS, 2 mM glutamine, 100 U/mL penicillin, and 100 μg/mL streptomycin. Twenty-four hours after re-plating, the original medium of the cells was aspirated off and fresh medium was added. Dilutions of achillolide A first in DMSO and then in the growth medium were made fresh from a stock solution just prior to each experiment and used immediately. The final concentration of DMSO in the medium was 0.2%. Dilutions of H_2_O_2_ in the growth medium were made freshly from 30% stock solution just prior to each experiment and were used immediately. Each treatment was performed in quadruplicate.

### 4.6. Determination of Cell Viability

Astrocytes were re-plated on 24-well PDL-coated plastic plates at a density of 1 × 10^5^/well, in DMEM *w*/*o* Phenol Red containing 2% FBS, 2 mM glutamine, 100 U/mL penicillin, and 100 μg/mL streptomycin. H_2_O_2_ and/or achillolide A were added, and cell viability was determined in 100 μL of cell supernatant using a commercial colorimetric assay (Roche Applied Science, Germany) according to the manufacturer’s instructions. This assay is based on the measurement of Lactate Dehydrogenase (LDH) activity released from the cytosol of damaged cells into the incubation medium. The absorbance was measured at 492 nm in a plate reader. The percentage of cytotoxicity was calculated according to the following equation:

$$Cytotoxicity\;\left(\%\right)=\frac{(A_{treated\;cells}-A_{untreated\;cells})\times 100}{A_{Triton-X\;treated\;cells}-A_{untreated\;cells}}$$

where the LDH activity in wells with untreated cells represents spontaneous cell death, and the *A_Triton-x treated cells_* is the maximum releasable LDH from the cells. In order to determine the maximum releasable LDH, untreated cells were lysed by the addition of 2% Triton x-100 (5 min, 37 °C) to the wells.

### 4.7. Enzyme-Linked Immunosorbent Assays (ELISA) for Total and Phosphorylated-MEK1 and p44/42 MAPK

Astrocytes were re-plated on six-well PDL-coated plastic plates at a density of 2 × 10^6^/well, in DMEM *w*/*o* Phenol Red containing 2% FBS, 2 mM glutamine, 100 U/mL penicilin, and 100 μg/mL streptomycin. Astrocytes were treated with achillolide A or memantine 40 min before the addition of H_2_O_2_. Cells were lysed in a lysis buffer supplied by PathScan sandwich ELISA kit (Cell Signaling Technology; Beverly, MA, USA) according to the manufacturer’s protocol. Protein concentration in cell lysates were determined by Bradford reagent (Bio-Rad, Hercules, CA, USA), and equal amounts of proteins were subjected to ELISA. To measure the amount of total and phosphorylated MEK1 in cell lysates of astrocytes, ELISA was performed according to the manufacturer’s protocol using the PathScan total MEK1 sandwich ELISA kit (Cell Signaling Technology) and the PathScan phosoho-MEK1 (Ser217/221) sandwich ELISA kit (Cell Signaling Technology), respectively. To measure the amount of total and phospho-p44/42 MAPK in cell lysates of astrocytes, ELISA was performed according to the manufacturer’s protocol using the PathScan phosoho-p44/42 MAPK (Thr202/Tyr204) sandwich ELISA kit (Cell Signaling Technology), or the PathScan total p44/42 MAPK sandwich ELISA kit (Cell Signaling Technology), respectively. The optical density was determined at 450 nm using a microplate reader.

### 4.8. Evaluation of Intracellular ROS Levels

Intracellular ROS levels were detected using the non-fluorescent cell permeating compound, 2′7′-dichlorofluorescein diacetate (DCF-DA). DCF-DA is hydrolyzed by intracellular esterases and then oxidized by ROS to a fluorescent compound 2′-7′-DCF. Astrocytes were re-plated onto 24 wells poly-d-lysine-coated (PDL) plastic plates (300,000 cells/well) and treated with DCF-DA (20 μM) for 30 min at 37 °C. Following incubation with DCF, cultures were rinsed twice with PBS and then re-suspended in DMEM containing 10% FBS, 8.4 mM HEPES, 2 mM glutamine, 100 U/mL penicillin, and 100 μg/mL streptomycin. ROS levels (fluorescence) at time 0 were measured in a plate reader with excitation at 485 nm and emission at 520 nm. Astrocytes were then treated with achillolide A for 2 h before the addition of H_2_O_2_ and ROS levels (fluorescence) were measured in a plate reader with excitation at 485 nm and emission at 520 nm after 1 h. The percentage of ROS levels was calculated according to the following equation (where F is the fluorescence):

$$ROS\;levels\;\left(\%\right)=\frac{\left(F_{achillolide\;\&\;H_{2}O_{2}\;treated\;cells}-F_{untreated\;cells}\right)\times 100}{F_{H_{2}O_{2}\;treated\;cells}-F_{untreated\;cells}}$$

### 4.9. Determination of H_2_O_2_ Scavenging Activity

The scavenging of H_2_O_2_ was determined by the method of Ruch *et al.* [59], using 1 mM instead of 4 mM H_2_O_2_. H_2_O_2_ solution (1 mM H_2_O_2_ in PBS) was incubated with different concentrations of achillolide A, quercetin, or memantine. Absorbance (A230) was determined spectrophotometrically 10 min later against blank solutions containing achillolide A, quercetin, or memantine in PBS without H_2_O_2_.

### 4.10. Diffrential Pulse Voltammetry (DPV)

Fifteen point four microliters of achillolide A stock solution (78 mM in DMSO) were added to 484.6 μL of 100 mM Phosphate Buffer Saline (PBS) pH = 7.4, yielding an 250 μM sample for analysis. Five microliters of the memantine stock solution (25 mM in DDW) were added to 495 μL of 100 mM Phosphate Buffer Saline (PBS) pH = 7.4, yielding a 250-μM sample for analysis. The samples were placed in a cyclic voltammeter cell, equipped with a working electrode (3.2 mm in diameters, glassy carbon), a reference electrode (Ag/AgCl), and an auxiliary electrode (platinum wire). Activation of the working electrode potential was done at a scan rate of 40 mV/s, pulse amplitude of 50 mV, sample width of 16 ms, pulse width of 50 ms, and pulse period of 200 ms. The output of the DPV experiments was a potential-current curve. An electrochemical working station (CH Instruments Inc. 610B, Austin, TX, USA) was used. During operation of the DPV, a potential current curve was recorded [52]. In general, the anodic potential represents the identity of the reducing equivalent or of the group of reducing equivalents and is expressed in volts. The current (I1 and I2) measured at each peak potential (E1 and E2) correlates with the reducing equivalent concentration according to the Randles–Sevcik equation [Ip = 2.69 × 105n3/2AD1/2Cn1/2] [60].

### 4.11. Statistical Analysis

Statistical analyses were performed with one-way ANOVA followed by Tukey-Kramer multiple comparison tests using Graph Pad InStat 3 for windows (GraphPad Software, San Diego, CA, USA).

## 5. Conclusions

Achillolide A, a sesquiterpene lactone which was isolated from *Achillea fragrantissima*, protects astrocytes against oxidative stress-induced cell death by reducing intracellular reactive oxygen species and interfering with MAP kinases activation. 

## Abbreviations

The following abbreviations are used in this manuscript:

DPV: Differential pulse voltammetry;
MAPK: Mitogen-activated protein kinases;
MEK: MAP/ERK kinases;
ROS: Reactive oxygen species.
